# Human Disease Phenotypes Associated with Loss and Gain of Function Mutations in *STAT2*: Viral Susceptibility and Type I Interferonopathy

**DOI:** 10.1007/s10875-021-01118-z

**Published:** 2021-08-26

**Authors:** Christopher James Arthur Duncan, Sophie Hambleton

**Affiliations:** 1 Translational and Clinical Research Institute, Immunity and Inflammation Theme, Newcastle University, Newcastle upon Tyne, NE2 4HH UK; 2 Royal Victoria Infirmary, The Newcastle Upon Tyne Hospitals NHS Foundation Trust, NE1 4LP Newcastle upon Tyne, UK; 3 Great North Children’s Hospital, The Newcastle Upon Tyne Hospitals NHS Foundation Trust, NE1 4LP Newcastle upon Tyne, UK

**Keywords:** Signal transducer and activator of transcription 2, antiviral immunity, type I interferonopathies, inborn errors of immunity, type I interferon, interferon-alpha/beta/lambda

## Abstract

STAT2 is distinguished from other STAT family members by its exclusive involvement in type I and III interferon (IFN-I/III) signaling pathways, and its unique behavior as both positive and negative regulator of IFN-I signaling. The clinical relevance of these opposing STAT2 functions is exemplified by monogenic diseases of STAT2. Autosomal recessive STAT2 deficiency results in heightened susceptibility to severe and/or recurrent viral disease, whereas homozygous missense substitution of the STAT2-R148 residue is associated with severe type I interferonopathy due to loss of STAT2 negative regulation. Here we review the clinical presentation, pathogenesis, and management of these disorders of STAT2.

## Introduction

Inborn errors of immunity are important not only in their own right as serious human diseases, but for what they teach us about the action and regulation of pathways of human immunity. Over the last two decades, a range of monogenic disorders have been identified that impact, in opposing ways, the activity of the antiviral type I and III interferon (IFN-I/III) systems. The clinical consequence of these defects (reviewed elsewhere [1–3]) includes (i) susceptibility to severe viral disease, including pandemic SARS-CoV-2 [4], due to inadequate IFN-I/III activity; or (ii) a spectrum of autoinflammatory disease phenotypes, collectively termed type I interferonopathies, associated with excessive IFN-I activity. This knowledge has led to a greater appreciation of the protective and pathogenic effects of IFNs in humans. Mendelian disorders of the transcription factor STAT2 have contributed significantly to our understanding of these dual roles of IFN-I in antiviral defense and immunopathology, by underlining the unique function of STAT2 as both positive and negative regulator of IFN-I activity.

## STAT2

The principal role of STAT2 is to transduce signals downstream of the receptors for IFN-I and IFN-III. STAT2 was identified by the labs of Darnell and Stark, in elucidating the molecular pathways governing the response to IFN-I. Key discoveries were the identification of an interferon-stimulated response element (ISRE) in DNA [5] that was bound by a polyprotein complex termed interferon-stimulated gene factor 3 (ISGF3) [6]. ISGF3 included a 113-kDa protein, subsequently shown to be the product of the *STAT2* gene [7]. Parallel mutagenesis studies in the human fibrosarcoma cell line U6A identified STAT2 to be an essential activator of gene transcription in response to IFN-I but not IFNγ [8]. The relevance of STAT2 to antiviral immunity was subsequently confirmed by generation of STAT2 knockout (*Stat2* − / −) mice [9], which showed impaired responses to IFN-I and were susceptible to vesicular stomatitis virus (VSV) infection, similar to *Stat1* − / − [10] or *Ifnar1* − / − [11] mice.

## STAT2 Signaling

**Fig. 1:**
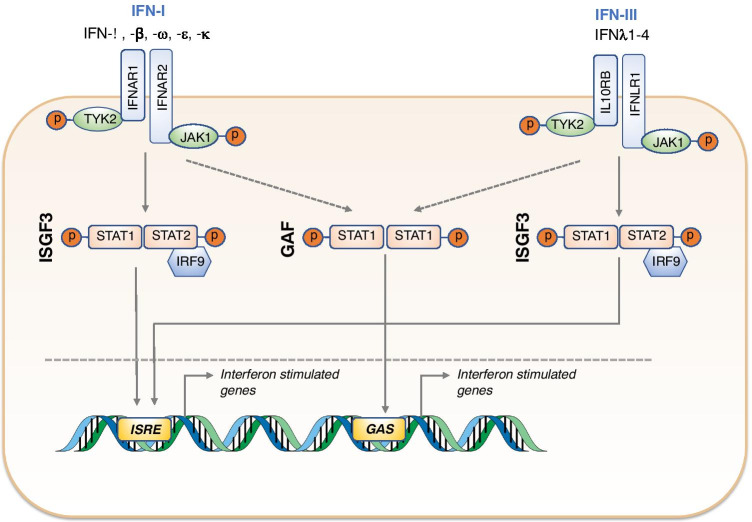
Function of STAT2 within IFN-I and IFN-III pathways. STAT2 plays a role in induction of interferon-stimulated genes (ISGs) through its involvement in interferon-stimulated gene factor 3 (ISGF3)

These seminal studies led to the canonical model of STAT2 signaling summarized in Fig. 1. In this model, STAT2 is activated by tyrosine phosphorylation through the action of receptor-associated kinases JAK1 [12] and TYK2 [13]. It associates with tyrosine phosphorylated STAT1 and IRF9 in a heterotrimeric transcription factor complex known as interferon-stimulated gene factor 3 (ISGF3). ISGF3 translocates to the nucleus, binding to ISRE in the promoters of hundreds of interferon-stimulated genes (ISGs). A small contribution to the antiviral response is also made by tyrosine phosphorylated homodimers of STAT1, which bind to a separate motif known as the gamma activated sequence (GAS) [14].

While this is a useful conceptual model, findings over the last few years indicate a more complex reality. For instance, STAT2 participates in transcriptionally active complexes other than ISGF3. These “noncanonical” complexes include STAT2:IRF9 [15] and an unphosphorylated form of ISGF3 [16, 17]. The topic of noncanonical STAT2 signaling has recently been reviewed [18]. A recent study also challenges the notion that ISGF3 assembles in the cytosol, as in the canonical model, suggesting instead that uSTAT2:IRF9 is bound to DNA under homeostatic conditions, where it governs basal transcription, and is subsequently displaced by ISGF3 upon IFN-I treatment [15]. Regardless of the precise mode of action of STAT2, its importance to human antiviral immunity has been revealed by the discovery of humans with homozygous STAT2 deficiency, who exhibited a clinical phenotype of increased susceptibility to various viruses [19–23]. Clinical aspects of this disorder will be considered in more detail in a later section.

## STAT2 Structure and Interactions

**Fig. 2:**
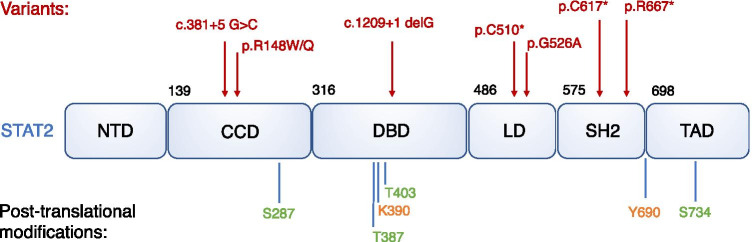
Model of STAT2. Demonstrating domains, disease-causing variants in STAT2 (in red) and known post-translational modifications (in green = negative regulation of transcriptional activity, in orange = positive regulation of transcriptional activity)

In common with other members of the STAT family, STAT2 has six functional domains—the N terminal domain (NTD), coiled coil domain (CCD), DNA binding domain (DBD), linker domain (LD), Src homology 2 (SH2) domain, and the C-terminal transactivation domain (TAD; Fig. 2).

The activity of STAT2 is regulated by post-translational modification. The most well-known is phosphorylation of Y690 which enhances its transcriptional activity. Mutagenesis studies have identified that phosphorylation of additional residues (S287 [24], T387 [25], S734 [26]) on STAT2 negatively regulates its transcriptional activity. STAT2 T387 is constitutively phosphorylated, negatively regulating ISGF3 binding to DNA; constitutive phosphorylation at T403 maintains STAT2 dimerization with STAT1 [27]. By contrast, IFN-mediated acetylation of STAT2 K390 promotes transcription [28] (Fig. 2).

Unphosphorylated STAT2 shuttles continuously between the cytosol and the nucleus, but owing to a strong nuclear export signal (NES) is predominantly cytosolic [29]. Here, STAT2 associates with various proteins including IRF9 [30, 31], STAT1 [32, 33], and IFNAR2 [34]. The interactions with STAT1 and IRF9 are mediated by the NTD and CCD of STAT2 respectively. Interestingly, although STAT2:IRF9 or STAT1:STAT2 complexes can be readily identified, there is limited evidence that STAT1 and IRF9 directly interact [15, 31]. The interaction domain with IFNAR2 has not been precisely mapped, but involves the proximal third (1–315) of STAT2, incorporating the NTD and CCD [34]. Upon phosphorylation at Y690, STAT2 rapidly accumulates in the nucleus due to a conformational change that impedes accessibility of the STAT2 NES [29].

Certain aspects of STAT2 structure and function distinguish it from other STATs. For example, STAT2 does not bind directly to DNA, as it lacks a strong DNA binding domain (it relies on STAT1 and/or IRF9 to bind to DNA) [35]. STAT2 makes an essential contribution to ISGF3 function by recruiting transcriptional coactivators through its TAD [35, 36]. It also exhibits the most interspecies divergence of all STAT molecules, particularly in the TAD [37]. Nevertheless, mouse STAT2 TAD can complement human STAT2 activity [38, 39].

## Viral Targeting of STAT2

Selection pressure exerted by viruses has been proposed as an explanation for the increased sequence diversity of STAT2 [37]. Reflecting the critical role of STAT2 in the antiviral IFN response [9, 19], certain viruses target STAT2 for degradation as an IFN evasion strategy. Examples are flaviviruses (dengue, Zika, and hepatitis C virus) [40–42], paramyxoviruses (parainfluenza virus 2 and respiratory syncytial virus) [43–45], and herpesviruses (CMV) [46]. There is evidence that the sequence diversity of STAT2 restricts interspecies viral transmission, in that it impacts the ability of certain viral proteins to bind and degrade STAT2 of other species [40, 41, 47]. The result is that certain viral pathogens only cause disease in humans. Examples include dengue [40] and Zika [41] virus and human metapneumovirus [48].

## Regulatory STAT2 Functions

Beyond its importance to antiviral immunity, in recent years STAT2 has been shown to participate in regulation of immune signaling. Functions recently identified include cross-regulation of STAT1 [32] and NF-KB signaling [49], as well as negative regulation of IFNAR signaling [50], via the ubiquitin-specific protease 18 (USP18).

STAT2 was shown to cooperate with the NF-KB pathway to positively regulate the induction of the *IL6* gene [49]. IL6 is an important proinflammatory cytokine. Nan and colleagues showed that when STAT2 and IRF9 expression was increased, the uSTAT2:IRF9 complex interacted with p65, bridging the *ISRE* and *KB* elements in the *IL6* promoter, leading to the enhanced expression of *IL6* in response to IL-1B, tumor necrosis factor, or lipopolysaccharide (LPS) [49].

STAT2 also has negative regulatory activity toward cytokine signaling pathways. In resting cells, STAT2 and STAT1 bind strongly [32], with the net effect of retaining STAT1 in the cytosol via the dominant activity of the STAT2 NES (discussed above). Upon exposure to cytokines that activate STAT1 but not STAT2 (e.g., IFNγ, IL27), the interaction inhibits pSTAT1 from forming homodimers and participating in signaling [32]. Thus, in the absence of STAT2, the transcriptional output of these cytokines becomes dominated by STAT1 [32]. Loss of this regulatory function may contribute to certain inflammatory manifestations of STAT2 deficiency [20–22], described below.

STAT2 has also been recently shown to participate in negative feedback toward IFN-I signaling [50], where it supports the activity of a key negative regulator, USP18 [51, 52]. USP18 is an essential regulator of IFN-I signaling, as revealed by the profound pathological consequences for USP18-deficient humans and mice [53–55]. The precise details of STAT2’s role in supporting USP18-mediated regulation will be discussed in more detail below. The clinical importance of this latter function of STAT2 was recently confirmed by the discovery of children with fatal IFN-I-mediated inflammatory disease attributed to homozygous missense mutations of *STAT2* [56, 57].

## Inborn Errors of Immunity Caused by Mutations in STAT2

### Autosomal Recessive STAT2 Deficiency

#### Genetics

The human *STAT2* gene is found on chromosome 12 and contains 24 exons. Homozygous or compound heterozygous variants in *STAT2* leading to complete deficiency of STAT2 protein have been identified in 11 individuals in five kindreds [19–22, 46]. Five distinct loss of expression variants have been reported, resulting in either frameshift or splicing defects leading to nonsense mediated RNA decay. Heterozygous carriers of these variants appear clinically unaffected. Mutations associated with complete STAT2 deficiency and the associated clinical phenotypes are summarized in Table 1.

**Table 1:** Clinical features of autosomal recessive STAT2 deficiency

Variant	LAV dissemination	Severe/recurrent viral disease	Uncomplicated infections	Hyperinflammation	Ref
c.381 + 5 G > C (splicing defect)	P1—MMR pneumonitis/hepatitis P2—unvaccinated P3—vaccine history unknown P4—SNHL post-MMR* P5—unvaccinated	P1—HSV1 gingivostomatitis - IAV pneumonia P2—Fatal viral illness (10w) P3—none noted (childhood history unknown) P4—bronchiolitis P5—hospitalization with viral illness	P1—none reported P2—none reported P3—none reported P4—varicella P5—varicella	None reported	[19]
c.1836 C > A, p.C617*	P1—acute febrile illness post-MMR* P2—acute febrile illness with MuV in CSF	P1—Opsoclonus-myoclonus syndrome post-MMR with CSF lymphocytosis Recurrent opsoclonus-myoclonus with meningoencephalitis P2—None reported	P1—not reported P2—not reported	P1—critical illness with pancytopenia in context of meningoencephalitis P2 – “septic shock,” organism not identified	[20]
c.1528 C > T, p.R510* and c.1576 G > A, p.G526A (splicing defect)	P1—MMR rash and hepatitis* P2—MMR pneumonitis/hepatitis with coagulopathy	P1—severe RSV, EV, AdV - fatal febrile illness with DIC (7y), organism not identified (viruses not tested) P2—severe recurrent varicella - EV meningitis - prolonged primary EBV	P1—none reported P2—RSV, IAV, EV, AdV HPV molluscum	P1—recurrent severe febrile episodes P2 – “inflammatory” responses to viral infection with cytopenia and T cell activation	[21]
c.1209 + 1delG (splicing defect)	P1—acute febrile illness with MuV in CSF	RSV, norovirus, EV leading to hospitalization	None reported	HLH secondary to MMR	[22]
c.1999 C > T, p.667*	Acute febrile illness post-MMRV with probable post vVZV varicella*	Life-threatening IAV pneumonitis Febrile seizure with CoV HKU1 infection Rhinovirus pneumonia	Recurrent rhinovirus, PIV3, HMPV	HLH secondary to MMR	[23]

^*^Vaccine-strain virus either not tested or not confirmed

*AdV*, adenovirus; *BCG*, Bacille Calmette-Guerin; *CMV*, cytomegalovirus; *CNS*, central nervous system; *EV*, enterovirus; *HHV6*, human herpesvirus 6; *HLH*, hemophagocytic lymphohistiocytosis; *HMPV*, human metapneumovirus; *HPV*, human papillomavirus; *HRV*, human rhinovirus; *HSV*, herpes simplex virus; *IAV/IBV*, influenza A/B virus; *LPD*, lymphoproliferative disease; *MMR*, measles, mumps, and rubella vaccine; *MuV*, mumps virus; *PIV*, parainfluenza virus; *RSV*, respiratory syncytial virus; *SNHL*, sensorineural hearing loss; *VZV*, varicella zoster virus; *vVZV*, varicella zoster virus vaccine

#### Viral Susceptibility

The primary manifestation of autosomal recessive (AR) STAT2 deficiency is susceptibility to severe and/or recurrent viral disease in individuals without other clinical or laboratory evidence of immunodeficiency. A particularly striking aspect of this phenotype is susceptibility to disease caused by live-attenuated viral (LAV) vaccines, such as measles, mumps, and rubella (MMR) or varicella zoster virus (vVZV). This is in common with other monogenic defects of IFN-I/III immunity (reviewed in [3]). Of the eight STAT2-deficient individuals known to have been exposed to LAV vaccines, all developed viral illness in temporal association. Vaccine-strain viral dissemination was confirmed by PCR in 4/8 cases (in the others, testing was either not done or not reported). One STAT2-deficient individual was identified in adulthood by family screening [19]. The expectation is that she would have been in receipt of measles vaccine in childhood, and had antibodies to measles consistent with exposure to either wild-type virus or vaccine [19]. Thus, susceptibility to LAV vaccines may not be fully penetrant in STAT2-deficient patients. Problems handling other LAV vaccines (such as the yellow fever vaccine) have not been reported in STAT2 deficiency, but would be expected by analogy to homozygous IFNAR1 deficiency [58] or IFN-I autoantibodies [59].

In addition to susceptibility to LAV vaccines, which serves as a “red flag” for defects of IFN-I/III immunity (reviewed in [3]), STAT2-deficient patients also experience increased susceptibility to naturally acquired viral disease. This includes a range of DNA and RNA viruses acquired at the mucosal surface, including influenza virus, enteroviruses, EBV, and adenovirus (Table 1), presumably due to the involvement of STAT2 in both IFN-I and IFN-III signaling pathways [3]. The penetrance of this phenotype is more variable, ranging from death in early infancy or childhood to survival into adulthood with no obvious phenotype [19], recalling the incomplete clinical penetrance of several other inborn errors of innate immunity [60–62]. Also in common with such disorders, [63, 64], and despite limited follow-up of STAT2 deficiency to date, there seems to be a reduction in severity of infections over time [19, 22, 23], which might point to the maturation of compensatory adaptive immunity [62]. STAT2-deficient individuals generally have normal laboratory indices of adaptive immunity, and mount appropriate serological responses to vaccination.

STAT2-deficient patient cells demonstrate defects of ISG expression and induction of the antiviral state in response to IFN-I, which can be rescued by *STAT2* complementation. This defect can also be overcome in vitro by treatment with IFNγ [21]. Whether IFNγ might offer an option for antiviral therapy in patients with STAT2 deficiency has not been tested. In part, this may be due to concerns that use of IFNγ during acute viral disease might exacerbate the hyperinflammatory state that can accompany viral disease in STAT2 deficiency.

#### Hyperinflammation

Hyperinflammatory features such as prolonged fevers requiring hospitalization in response to viral infection [20, 21], unprovoked sepsis-like presentations [20, 21], and even HLH [22, 23] have been noted in approximately two-thirds of patients with AR STAT2 deficiency (Table 1). The pathogenesis is unknown, and may be multifactorial. In most cases, hyperinflammation occurred in the context of viral infection or live-attenuated viral vaccination, implying that viral infection is a trigger. However, the occurrence of cases of hyperinflammation without convincing evidence of viral infection [20] raises the possibility of a more complex defect of STAT2-dependent immunoregulation. This is not surprising, considering the emerging evidence for immunoregulatory functions of STAT2.

As in patients, STAT2-deficient mice exhibit inflammatory phenotypes. *Stat2* − / − mice are prone to hyperinflammation and macrophage activation following influenza infection [65]. This hyperinflammatory state was able to confer protection against bacterial superinfection. Furthermore, *Stat2* − */* − mice are more susceptible to endotoxic shock [66], in contrast to *Ifnar1* − / − mice which are protected [67]. The mechanism(s) underlying these phenomena have not been elucidated. Deletion of STAT2 in murine macrophages has been shown to alter their cellular response to inflammatory signals. *Stat2* − / − macrophages express MHC class II in response to IFN-I, through a mechanism involving IRF1 and STAT1 [68], whereas in WT macrophages MHC class II is typically induced by IFNγ. Thus, loss of STAT2 seemingly alters the transcriptional response to IFN-I, potentially with inflammatory consequences. Whether this is also true in humans has yet to be established. Considering the regulatory functions of STAT2 described later in this review, it is conceivable that STAT2 loss may have more complex effects on immunoregulation. Further studies are warranted to explore the immunological basis of hyperinflammation in STAT2 deficiency.

#### Diagnosis and Management

Diagnosis of STAT2 deficiency relies on a high index of clinical suspicion and is confirmed by genetic testing. Although not clinically validated, laboratory screening approaches prior to genetic testing may increase the yield. Such assays include analysis of IFN-I signaling activity and/or STAT2 protein expression by immunoblot [19, 23].

Much remains to be learned about the optimal clinical management of STAT2 deficiency. While LAV vaccines (e.g., MMR, varicella, yellow fever) should be avoided, other inactivated and recombinant vaccines are strongly advised. Immunoglobulin supplementation has been proposed as a therapy in STAT2 deficiency, and was associated with a reduction in the frequency of infections and episodes of inflammation in two cases in which it was used [21, 22]. Owing to the range of clinical expressivity noted in STAT2-deficient kindreds, where it is clear that some STAT2-deficient patients live into adulthood with no apparent disease phenotype, there remains a case for individualized therapeutic decision-making.

Management of hyperinflammation in AR STAT2 deficiency is similarly an evolving area, requiring further mechanistic studies to understand its pathophysiology. As mentioned, IVIG may have a role in acute management of hyperinflammatory episodes [21, 22], as it does in other inflammatory syndromes [69]. Its mechanism of action in this context is unclear. There may also be a role for other immunomodulators, by analogy to COVID-19 in adults [70, 71]. However, episodes of hyperinflammation have also been reported to resolve with conservative management [23].

Hematopoietic stem cell transplantation (HSCT) has not been undertaken to date in AR STAT2 deficiency, unlike AR STAT1 deficiency [72]. The latter, in addition to conferring a profound defect of IFN-I/III signaling, critically disables IFNγ signaling between cells of the immune system which are replaced during allogeneic HSCT. In contrast, the viral susceptibility of STAT2 deficiency probably results from impaired IFN-I/III signaling in non-hematopoietic tissues, untouched by HSCT. Furthermore, the transplant process is inevitably accompanied by a temporary loss of adaptive immune protection against viral infection that would be particularly hazardous in the context of impaired innate immunity. This does not necessarily preclude a possible role for HSCT in special circumstances, for example, severe/recurrent treatment-refractory HLH.

### Autosomal Recessive STAT2-Associated Type I Interferonopathy (STAT2 Gain of Function)

#### Genetics

Homozygous missense variants affecting the same arginine 148 residue of STAT2 have been identified in three children in two kindreds with severe early-onset type I interferonopathy [56, 57]. Heterozygous carriers of these variants appear clinically unaffected. The mutations and their associated phenotypes are summarized in Table 2. Type I interferonopathy is a term used to describe a group of Mendelian diseases characterized by neurological and multisystem disease associated with increased IFN-I activity in blood and cerebrospinal fluid (reviewed in [73]). Aicardi-Goutières syndrome [74], which phenocopies congenital viral infection, is a prototypical type I interferonopathy.

**Table 2:** Clinical features of autosomal recessive STAT2–associated type I interferonopathy (STAT2 gain of function)

Gene/variant	Neurological features	Inflammatory features	Other features	Response to ruxolitinib	Outcome	Ref
*STAT2* c.442 C > T p.R148W	P1—Seizures Intracranial calcification Hemorrhage White matter changes P2—Abnormal EEG Intracranial calcification Hemorrhage White matter changes Cerebellar hypoplasia BS atrophy	P1—Neonatal sepsis Recurrent HLH-like inflammation P2—None reported	P1—TCP TMA Proteinuria Preterm birth P2—TCP Preterm birth Recurrent apnoea	P1—Yes (2.5 mg b.d.) P2—Partial (1 mg b.d.—Improved ISG score but persistent neurodevelopmental abnormalities)	P1—Died in immediate post HSCT period (20 months) P2—Died (3 months)	[56]
*STAT2* c.443 G > A p.R148Q	Seizures Intracranial calcification	Fever	Adenitis Cardiomegaly ILD Respiratory failure	N/A	Died (5 months)	[57]
*USP18* P1–3: c.652C > T, p.Gln218* P4–5 c.652C > T (het) with large cryptic 3ʹ deletion (het)	P1—Microcephaly Intracranial calcification Cortical destruction P2—Abnormal EEG Hemorrhage P3—Seizures Hemorrhage Cortical necrosis White matter changes P4—Seizures Intracranial calcification Massive hemorrhage Enlarged lateral ventricles Cerebellar hypoplasia Malformation of BS and PF P5—Seizures Hemorrhage Abnormal cortical gyration Cysts	P1—NA P2—None reported P3—None reported P4—None reported P5—None reported	P1—NA P2—TCP PDA Liver dysfunction P3—Ascites Abn renal appearances ASD, PDA Liver dysfunction P4—TCP Dyserythropoiesis Ectopic calcifications P5—TCP Ectopic calcifications Hepatomegaly Pleural effusions	N/A	P1—TOP (22w) P2—Died (7d) P3—Died (17d) P4—Died (22d) P5—Died (12d)	[54]
*USP18* c.1073 + 1 G > A—leading to deletion of exon 10	Seizures Hydrocephalus Intracranial calcification Intracerebral hemorrhage White matter changes	Fever Cellulitis	Shock with ARDS Necrotising cellulitis	Yes (5–10 mg b.d.)	Developmental delay but otherwise well (3 years)	[55]

Features of autosomal recessive USP18 deficiency are also included for reference

*ARDS*, acute respiratory distress syndrome; *BS*, brainstem; *EEG*, electroencephalogram; *HSCT*, hematopoietic stem cell transplantation; *ILD*, interstitial lung disease; *ISG*, interferon-stimulated gene; *PDA*, patent ductus arteriosus; *PF*, posterior fossa; *TCP*, thrombocytopenia; *TMA*, thrombotic microangiopathy; *TOP*, termination of pregnancy

#### Clinical Phenotype

STAT2-associated type I interferonopathy was originally reported in two brothers born of consanguineous parents of Pakistani origin, bearing a very rare homozygous missense substitution of arginine by tryptophan (R148W) in STAT2 [56]. The proband presented with recurrent episodes of sterile systemic inflammation, which met clinical diagnostic criteria for HLH, accompanied by neurological features suggestive of type I interferonopathy [73], such as seizures, intracranial calcifications, hemorrhages, cerebral white matter changes, and developmental regression. Investigations revealed transcriptional evidence of heightened IFN activity in whole blood. There was a clinical response to corticosteroids and the JAK inhibitor ruxolitinib; however, he died due to complications of HSCT. His younger brother was more severely affected, particularly from the neurological perspective, and despite treatment with ruxolitinib did not survive beyond early infancy. In parallel, another individual was reported with a similar clinical phenotype (including seizures and intracranial calcification) associated with a homozygous missense variant affecting the same residue of STAT2—in this case replacing arginine with glutamine (R148Q) [57]. This infant similarly had transcriptional evidence of increased IFN activity in whole blood. Additional features were fistulating adenitis and progressive lung disease which led to his death aged 5 months. There was a family history of infant death in two siblings with a similar clinical syndrome. In all three patients, the phenotype recalled USP18 deficiency [54, 55] as summarized in Table 2.

In cells from patients bearing STAT2 R148 variants, there was evidence of significantly enhanced late transcriptional responses to IFN-I [56, 57]. In the case of R148W cells, there was also a clear phenotype of prolonged phosphorylation of JAK1, STAT1, and STAT2 [56] indicative of a defect of negative regulation of IFNAR signaling upstream of STAT2, reminiscent of USP18 deficiency [54]. This phenotype was not observed in response to IFNγ [56, 57] or other cytokines [56] and thus was specific to IFN-I. Patient fibroblasts from patients [56] or reconstituted U6A cells [57] were insensitive to overexpression of *USP18*, while knockdown of *USP18* had no effect [56], indicating that USP18 function was impaired in the presence of R148W/Q variants. These findings indicated a defect of STAT2’s supportive function toward USP18 [50].

#### Molecular Pathogenesis

USP18 fulfills its negative feedback function by binding to IFNAR2, displacing JAK1 and altering the conformation of the IFN-IFNAR1-IFNAR2 complex [75]. This impedes JAK1 phosphorylation—an essential step in IFNAR signaling—and consequently blocks tyrosine phosphorylation of STAT1 and STAT2 [50, 52, 75]. *USP18* expression is induced by ISGF3 signaling and its regulatory activity continues for the duration of its expression. Thus, USP18 is primarily responsible [52, 76] for the phenomena recognized in IFN biology whereby cells, after IFN-I treatment, become refractory to further restimulation [77]. STAT2 is essential for this function of USP18 [50]. A key question is how the R148W/Q mutations impair this regulatory function of STAT2.

**Fig. 3:**
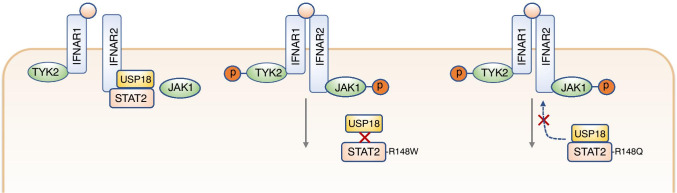
Models of STAT2-associated type I interferonopathy (STAT2 gain of function) pathogenesis. USP18 binds STAT2 and IFNAR2, displacing JAK1 from the cytoplasmic domain of IFNAR2 and inducing a conformational change in the IFN-IFNAR1-IFNAR2 complex, leading to impaired signal transduction (left). The R148W variant of STAT2 impairs interaction with USP18 (middle), whereas the R148Q variant demonstrates preserved interaction with USP18 but a defect of recruitment to IFNAR2 (right), leading to a defect of USP18-mediated negative feedback

USP18 is known to interact independently with both STAT2 and IFNAR2 via adjacent domains of USP18 [50]. In the current paradigm, STAT2 recruits USP18 to IFNAR2 (Fig. 3). The USP18:IFNAR2 interaction is substantially impaired (although not completely abolished) in the absence of STAT2 [50]. Consistent with this, deletion of the STAT2 binding site on IFNAR2 reduced the binding of USP18 to IFNAR2 [50]. The R148 residue is located in the CCD of STAT2, a region previously implicated in the interactions with both USP18 [50] and IFNAR2 [34]. Our studies demonstrated impaired interaction between STAT2-R148W and USP18, as measured by coimmunoprecipitation in U6A cells stably expressing WT or STAT2-R148W and treated with IFNα to induce USP18 expression [56] (Fig. 3). This was consistent with prior findings [50] and implied a defect of STAT2-dependent recruitment of USP18 to IFNAR2. Gruber and colleagues confirmed this in coimmunoprecipitation experiments in transiently transfected U6A cells overexpressing WT or STAT2-R148Q and USP18, showing a reduced interaction between USP18 and IFNAR2 in the presence of STAT2-R148Q (Fig. 3) [57]. However, the interactions between STAT2-R148Q and IFNAR2, as well as STAT2-R148Q and USP18, were preserved in coimmunoprecipitation experiments conducted in HEK293 and U6A cells [57]. Further work may resolve the precise molecular mechanism(s) underlying the impairment of USP18 activity in the context of STAT2 R148W/Q variants. Nevertheless, it is clear that the immunological and clinical impact of the loss of this regulatory activity of STAT2 is profound.

#### Diagnosis and Management

Clinical recognition of this very rare disease relies on awareness of the phenotype and is aided by identification of transcriptional evidence of elevated IFN activity in blood, as for other type I interferonopathies [73]. A suitable assay to rapidly screen for STAT2-R148W is to examine peripheral blood by phosflow for prolonged IFNα-induced phosphorylation of STAT1 and/or STAT2 [56]. Based on limited experience in STAT2-R148W [56] and USP18 deficiency [55], rapid initiation of JAK inhibitor treatment is a priority and can be life-saving. Interestingly, the clinical response to ruxolitinib was dose-dependent in USP18 deficiency, requiring doses above 5 mg b.d. to achieve clinical remission [55], although such doses are justified by the grave prognosis.

## Concluding Remarks

Molecular defects of STAT2 have already taught us much about the biological function of STAT2 in humans. One of the most interesting aspects is the relatively “mild” clinical phenotype of AR STAT2 deficiency, despite the extent of compromise to IFN-I and IFN-III systems. This presumably reflects both the capacity of viral pathogens to evade innate IFN restriction, and the residual ability of other facets of antiviral immunity to compensate. The variable clinical penetrance of AR STAT2 deficiency, even within the same kindred, is notable. Clearly, given the contribution of viral infection to the disease phenotype, pathogen exposure, infectious dose, and other virological factors are likely to be important determinants of penetrance. Indeed, penetrance is seemingly less variable in circumstances where exposure is more controlled, for example, in the context of administration of live-attenuated viral vaccine(s) to STAT2-deficient patients. In assessing individual patients, serological testing for prior exposure may be helpful to inform the extent of the vulnerability to naturally acquired viruses. A question for future studies is whether incomplete penetrance in STAT2 deficiency may also be governed by the effectiveness of adaptive immune compensation.

Disease associated with STAT2-R148Q has been termed STAT2 GOF. This terminology is convenient as it (i) helps to distinguish it from AR STAT2 deficiency and (ii) conforms to a well-recognized paradigm for other STATs (e.g., STAT1 or STAT3), where both LOF and GOF variants are recognized. However, mutations which lead to a gain of protein function that is pathogenic only in the homozygous state are exceedingly rare in nature [78, 79]. Indeed, GOF variants in STAT1/3 [80–83] or JAK1 [84, 85] manifest in the heterozygous state. In the case of STAT2, the GOF nomenclature is potentially misleading as it fails to account for the specific molecular defect of R148 STAT2, distinct from its role in ISGF3 [56]. From a protein-centric viewpoint, it is clear that STAT2-R148W/Q mutations impair the regulatory function of STAT2 toward USP18 [56, 57]. In other words, they cause a pathological *loss* of this particular STAT2 function.

**Fig. 4:**
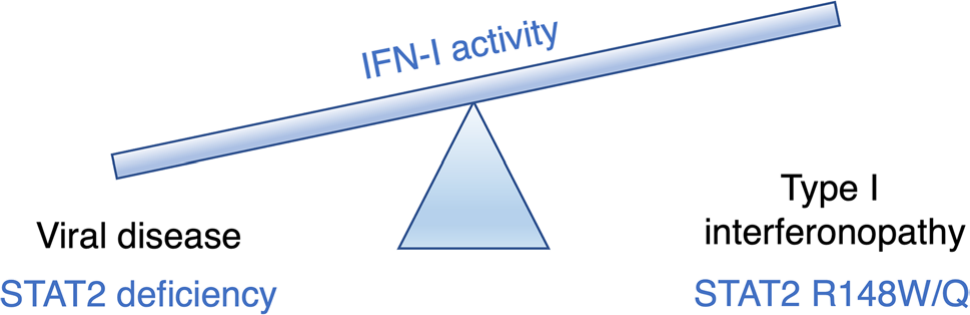
Summary of STAT2-associated disease phenotypes

The fascinating aspect of this disorder is that it underscores the unique role of STAT2 as both positive and negative regulator of IFN-I signaling pathway (Fig. 4). A key feature of STAT2 GOF (alongside similar defects such as USP18 deficiency) is its very early onset, apparently without an overt infectious precipitant, suggesting that physiological levels of IFNs produced during homeostasis are sufficient to initiate disease. It is conceivable that additional pathogenic variants in STAT2 might be identified in future that provide further insight into STAT2 biology. By analogy with STAT1 or STAT3 [80–83], we might predict autosomal dominant variants that confer a GOF of STAT2’s positive transcriptional activity, for example, by interfering with dephosphorylation of Y690, or via the loss of a regulatory residue such as T387. It is debatable whether partial LOF of STAT2, through hypomorphic AR or dominant negative AD inheritance, would be clinically manifest. The wider application of genomic sequencing methods, alongside in vitro and ex vivo measures of interferon signaling and viral susceptibility, will no doubt present opportunities to test these hypotheses in the coming years.

## Data Availability

Not applicable
